# Clonal hematopoiesis at the crossroads of Inflammaging and cardiovascular disease: Mechanistic insights and translational horizons

**DOI:** 10.46989/001c.146275

**Published:** 2025-12-23

**Authors:** Soumiya Nadar, Taha K. Dohadwala, Nitish Kumaresan, Shabbeer I. Ahamed, Sumia Fatima

**Affiliations:** 1 Faculty of Medicine, Tbilisi State Medical University, 0186, Tbilisi, Georgia; 2 Department of Medicine, David Tvildiani Medical University, 0159, Tbilisi, Georgia; 3 Department of Medicine, Rawalpindi Medical University, Rawalpindi, Pakistan; 4 Clinical Researcher, Clinical Trial Network, Houston, Texas, USA

**Keywords:** Clonal Hematopoiesis, Cardiovascular Diseases, Mutation, Inflammation, Risk Assessment

## Abstract

Clonal Hematopoiesis of indeterminate potential (CHIP) has been increasingly recognised as a risk factor for cardiovascular disease (CVD). Recent epidemiological and experimental studies have linked CHIP as an independent risk factor for myocardial infarction, stroke, and coronary artery disease, with specific mutations carrying a higher CVD risk. During the aging process, somatic mutations in genes, including DNMT3A and TET2, accumulate in the hematopoietic stem cells (HSCs), conferring both epigenetic and metabolic advantages that not only drive hematopoiesis towards a pro-inflammatory myeloid lineage but also reprogram innate immune cells, promoting a persistent inflammatory state. These myeloid derivatives, via increased IL-1β and IL-6 production, establish a pro-atherogenic environment and contribute to plaque instability, leading to an increased thrombotic risk and accelerated vascular aging. Although routine screening is not recommended for asymptomatic adults, targeted detection in high-risk individuals could benefit from preventive strategies. Incorporating CHIP into risk models may enable precision prevention, but prospective trials are needed to determine whether CHIP-guided interventions improve cardiovascular outcomes.

## Introduction

As we age, physiologically healthy tissues such as the skin,^1^ colon,^2^ esophagus,^3^ and blood^4^ acquire mutations in cancer-associated genes. In blood, this phenomenon is termed clonal hematopoiesis (CH).^4^ CH of indeterminate potential (CHIP) refers specifically to CH possessing somatic mutations in leukemia driver genes at a variant allele fraction (VAF) of ≥2% in the absence of diagnosed blood disorder or cytopenia.^5^

It is increasingly recognized as a systemic, age-associated risk factor for non-malignant diseases.^6,7^ CH-harbouring mutations in acute myeloid leukemia (AML)-associated genes are nearly ubiquitous (95%) in 50–70-year-olds.^8^ These are rarely seen before the age of 40, appearing in over 10% of those over 70, rising to 19.5% of those ≥90 years.^9,10^

CHIP most commonly involves mutations in epigenetic regulators such as DNMT3A, TET2, and ASXL1, as well as signaling genes like JAK2, with DNMT3A and TET2 being among the most frequently mutated genes. These ‘epigenetic gatekeepers’, whose somatic alterations confer a competitive proliferative advantage, lead to clonal expansion.^11–15^ These mutations, common in myelodysplastic syndrome (MDS) and AML, give hematopoietic stem cells (HSCs) a selective advantage, leading to clonal expansion detectable in blood.^16,17^

Studies have shown that mutated myeloid cells derived from CHIP clones exhibit a pro-inflammatory phenotype, with enhanced production of interleukin-1β (IL-1β) and interleukin-6 (IL-6), key mediators in the pathogenesis of atherosclerosis and vascular injury.^18,19^ This inflammatory axis links CHIP with the concept of “inflammaging,” a chronic, low-grade inflammatory state increasingly implicated in a variety of age-related diseases.^20^

The presence of CHIP has been associated with poorer outcomes after cardiovascular events, resistance to statin therapy, and increased thrombosis risk, particularly in individuals with JAK2 V617F mutations.^21^ Nonetheless, the recognition of CHIP as a modifiable contributor to cardiovascular disease (CVD) risk represents a significant shift in our understanding of cardiovascular pathophysiology. As aging populations grow and precision medicine advances, integrating CHIP status into cardiovascular risk models may offer new avenues for prevention and treatment.

## Genetic and Environmental Determinants of CHIP

The risk of developing CH is largely related to the stochastic acquisition of somatic mutations in HSCs during aging.^22^ However, environmental and genetic factors also play a role. It is more common in older men and smokers, and less common in Hispanics.^6,7^ Recent studies suggest genetic predispositions and the microbiome may influence its development and progression.^23,24^

Mutations in epigenetic regulators disrupt normal DNA methylation dynamics that govern HSC self-renewal and differentiation.^25^ These alterations alter the normal balance between stemness and lineage commitment, and expansion of CD150^high^ HSCs, while PRC2-mediated epigenetic “memory” may reinforce long-term dysfunction in HSC self-renewal and differentiation.^26^ As a result, the blood becomes enriched with monocytes and macrophages.^27^

CH increases the risk of CVD, including myocardial infarction (MI), stroke, CHD, and atherosclerosis independently of traditional risk factors.^16,28^ These CHIP-associated myeloid cells infiltrate the arterial wall and promote atherogenesis through multiple mechanisms. In murine models of CH, including those with Jak2^VF^ mutations, clonal expansion leads to increased macrophage proliferation and prominent formation of necrotic cores in atherosclerotic lesions.^29^ Similarly, TET2 downregulation has been associated with the formation and progression of atherosclerotic plaques,^30^ while DNMT3A deficiency alters the function of myeloid cells and promotes inflammation via the upregulation of specific cytokines and chemokines.^31^

HSCs reside in low-oxygen niches of the bone marrow and rely on aerobic glycolysis to minimize reactive oxygen species (ROS) and limit DNA damage.^32^ In CH, however, oxidative stress is elevated, potentially driving further mutations and telomere shortening. Excess ROS may trigger overproliferation of Hematopoietic Stem and Progenitor Cells (HSPCs), increasing cardiovascular risk even in the absence of driver mutations.^33^ Additionally, CH induces a pro-inflammatory environment that supports the expansion of mutated HSPCs. Chronic inflammation and oxidative stress reinforce one another, amplifying both clonal expansion and cardiovascular complications.^34^

CHIP not only increases cardiovascular risk but also interacts with metabolic risk factors, amplifying disease progression. Individuals with CHIP and elevated LDL cholesterol levels show a significantly heightened risk of atherosclerotic cardiovascular disease (ASCVD), a relationship not observed with other lipid variables or conventional risk factors. Serial coronary computed tomography angiographies (CCTAs) support this association by revealing early-stage atherosclerotic changes in CHIP carriers with high LDL.^35^

Additionally, CHIP has been linked to a higher incidence of type 2 diabetes.^36^ Biologically, CHIP mutations can lead to the production of more inflammatory macrophages. These are known to contribute to insulin resistance, a key factor in the development of type 2 diabetes. Elevated LDL cholesterol amplifies this inflammatory response, thus markedly amplifying diabetes risk.^36^ Mechanistically, TET2 mutations interfere with AMP-activated protein kinase (AMPK), a central regulator of cellular energy balance. This disruption can impair metabolic homeostasis and promote insulin resistance.^37^

## Mechanistic Pathways Linking CHIP to Cardiovascular Disease

Trained immunity refers to the long-term functional alteration of innate immune cells evoked by various endogenous danger signals, including damage-associated molecular patterns (DAMPs) released during cellular stress or tissue injury, or exogenous pathogenic conserved molecules such as pathogen-associated molecular patterns (PAMPs).^38^ The concept of trained immunity provides a unifying mechanism for these observations.

**Figure 1.**
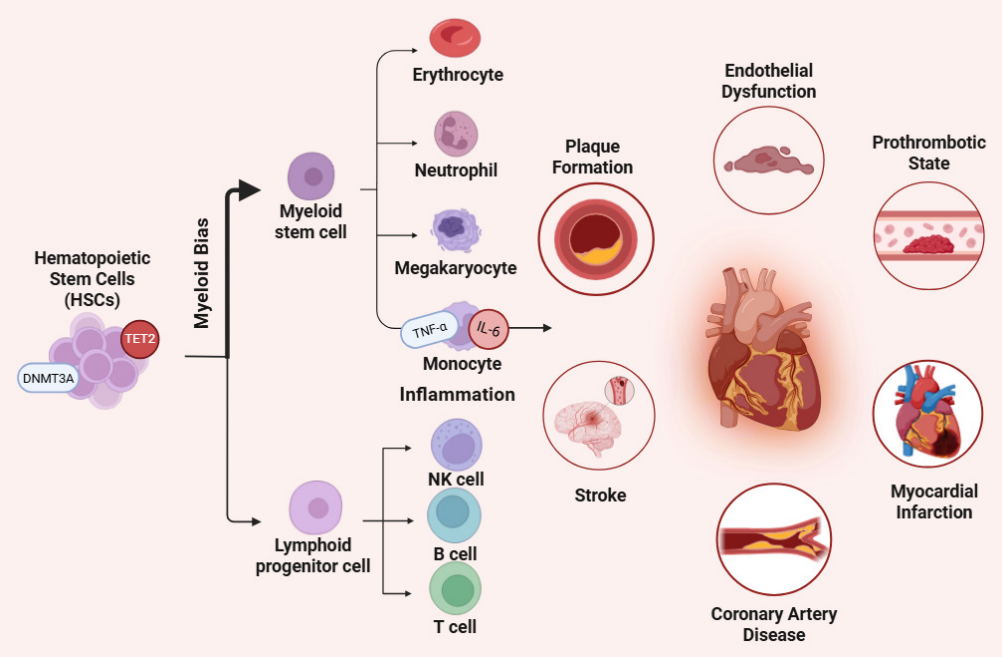
Schematic representation of clonal hematopoiesis-associated mutations (TET2, DNMT3A, ASXL1) reprogramming immune cell function and influencing checkpoint inhibitor response and toxicity. Created with BioRender.com

Insights from both human and animal studies have demonstrated that mutations associated with CHIP, particularly in TET2 and DNMT3A, reprogram innate immune cells such as monocytes and macrophages toward a persistently pro-inflammatory phenotype, thereby promoting atherosclerosis and other cardiovascular diseases^39,40^ (**Figure 1**). This link is reinforced by the transplantation of TET2-deficient bone marrow into atherosclerosis-prone mice, leading to an increase in plaque size, necrotic core formation, and vascular inflammation.^41^

While CHIP is classically defined by somatic mutations in HSCs, germline variants may also play a contributing role. Germline mutations in TET2 and DNMT3A are rare but have been described in familial cases of hematologic malignancy.^42^ Unlike somatic mutations, which are typically age-associated, germline variants present from birth may predispose individuals to earlier onset of CHIP and higher lifetime risk of both hematologic malignancy and CVD.^43^ Comparative studies suggest that germline and somatic variants share functional consequences such as disrupted DNA methylation, skewed myelopoiesis, and heightened inflammatory responses, but differ substantially in penetrance and phenotypic expression depending on gene dosage and environmental context.^44^

CHIP-associated leukocytes exhibit enhanced self-renewal, increased infiltration into atherosclerotic plaques, and amplified activation of the NLRP3 inflammasome.^18^ This results in elevated secretion of pro-inflammatory cytokines (IL-1β, IL-6, IL-18, and IL-8), matrix metalloproteinases (MMPs), and ROS. MMP-driven extracellular matrix degradation and oxidative stress from ROS contribute to plaque destabilization and rupture, thereby heightening thrombotic risk and accelerating vascular aging.^45^

In addition to the activation of inflammatory pathways during the formation of atherosclerosis, macrophages scavenge excessive lipid content. This becomes foam cells that lead to plaque formation and further impair the phagocytic capacity of these macrophages. In addition, upregulation of TET2 by CEBPA during transdifferentiation of pre-B cells to macrophages is required for upregulation of macrophage markers as well as phagocytic capacity, indicating a role for TET2 in regulating phagocytosis of macrophages.^39^

CHIP also acts synergistically with traditional CVD risk factors. In ApoE^−/−^ mice fed with a high-fat diet (20% fat, 0.15% cholesterol), TET2 overexpression reduced atherosclerotic lesions characterized by endothelial dysfunction, inflammation, and impaired autophagy.^30^ These vascular effects mirror those seen in CHIP, where TET2 deficiency exacerbates plaque burden, macrophage infiltration, and autophagy impairment, leading to unstable lesion phenotypes.

Observational studies link CHIP in atrial fibrillation to an increased stroke risk, suggesting that earlier or more intensive anticoagulation could be warranted in selected cases.^18^ In addition, the presence of CHIP in peripheral blood cells was associated with a 1.9 times increase in the risk of coronary heart disease (CHD) and 4 times the risk of MI in humans, and accelerated atherosclerosis in mice.^16^ Thus, CHIP not only enhances vascular inflammation but also compromises the integrity of atherosclerotic plaques, setting the stage for CVDs.

## Causality and Clinical Associations

CHIP is increasingly recognised as a risk factor for CVD, but whether it is a direct causal factor or simply a marker of aging and inflammation is still under investigation. Since CHIP prevalence sharply increases with aging,^9^ the possibility of confounding is substantial. Several prospective studies have expanded on this, adjusting for the traditional cardiovascular risk factors and found a strong independent association between CHIP and adverse cardiovascular outcomes. In a landmark trial that enrolled over 8000 patients, Jaiswal et al. demonstrated that CHIP-carriers face a 1.9-fold risk of coronary heart disease and a 4.0-fold increased risk of myocardial infarction (MI) compared to noncarriers. Furthermore, the trial identified specific mutations in DNMT3A, TET2, ASXL1, and JAK2 as independent contributors to heightened coronary heart disease risk. Moreover, mice with impaired TET2 in their bone marrow developed more severe atherosclerosis due to their macrophages showing increased expression of pro-inflammatory genes, highlighting the gene’s protective role against CVD by controlling inflammation.^16^

These results are supported in other large datasets. Using the UK Biobank, Bick et al.^46^ demonstrated that CHIP was associated with a 27% increased risk of CVD and a greater risk of 59% in larger CHIP clones (VAF > 10%) while Zuo et al.^47^ suggested that CHIP acts as an independent risk factor to cardiometabolic disease (CMD) progression in middle- and older-aged individuals.

To understand causality beyond observational studies, Mendelian randomization (MR) has provided compelling evidence linking CHIP-associated DNA methylation (Dnam) in genes to coronary artery disease (CAD) risk. Key mutated CHIP genes, DNMT3A and TET2, both epigenetic regulators, were found to have distinct but opposing Dnam patterns, contributing to the risk of CAD.^48^ On the other hand, the study by Jaiswal et al. utilised whole-exome sequencing in large human cohorts to associate specific CHIP gene mutations (DNMT3A, TET2, ASXL1, JAK2) with increased CAD risk, which was further strengthened through experimental in vivo studies in mouse models, which demonstrated that loss of TET2 function results in accelerated atherosclerosis.^16^ These studies establish CHIP as a causal factor in CAD through genetic studies, highlighting its role in CVD progression.

Despite these insights, various subgroups remain understudied. While CHIP among younger individuals is rare, the risk of CAD and other CVDs compared to age-matched non-carriers remains unknown. Moreover, mutation-specific analysis suggests heterogeneity in cardiovascular risk. For example, carriers with DNMT3A, TET2, and ASXL1 mutations had a 1.7-2.0 times risk of CAD compared to a 12.1 times risk of CAD in JAK2 V617F mutation carriers.^16^ Sex-based and ethnic differences in CHIP prevalence also remain unexplored.

Importantly, most data reflect population-level association. Integrating these findings into clinical practice requires individual risk modelling that incorporates mutation type, traditional risk factors, and inflammatory markers. The development of machine learning and longitudinal multi-omics integration may allow personalized prediction of CVD risk among CHIP carriers.

## Hematologic Implications and Transplant Considerations

Beyond its cardiovascular associations, CHIP has significant implications in hematology. Patients with CHIP have a 0.5–1% risk each year of developing hematologic malignancy, compared to <0.1% for people without CHIP.^6,9^ They are linked to a tenfold higher risk of hematologic cancers and increased all-cause mortality.^16,49^ The presence of CHIP mutations, particularly TP53, splicing factor genes, and IDH1/2, carries a higher risk of adverse outcomes and faster progression to overt hematologic malignancies.^50–52^

Donor CHIP is increasingly recognised as a clinically relevant factor in hematopoietic stem cell transplantation (HSCT). Rise in donor age is consistently associated with poor survival outcomes and increased treatment-related mortality.^53–55^ With the increasing prevalence of CHIP with age, CHIP-positive donors are relatively common among older donors, and transmission of CHIP clones to recipients could potentially result in donor-derived leukemia.^53,56^ Moreover, a recent study showed that CHIP-harboring donors had no effect on the overall survival but had an almost two-fold increased risk of chronic graft-versus-host disease (cGVHD).^57^

While CHIP is typically considered age-related and driven by random somatic mutations, emerging evidence suggests a genetic predisposition to it. CHIP is more frequent in individuals with first-degree relatives affected by myeloid malignancies, suggesting shared genetic susceptibility.^42^ However, twin studies show no significant difference in CHIP prevalence, indicating non-heritable factors may also play a major role.^58^ Excluding CHIP-positive donors when younger, mutation-free alternatives are available can reduce long-term morbidity and improve survival outcomes and post-transplant quality of life.^53^

Interestingly, not all donor CHIP confers poor outcomes, which has been supported by recent studies. Recipients of allogeneic HSCT from donors with CHIP showed a lower cumulative incidence of relapse/progression compared to CHIP-negative donors. This effect was especially more pronounced in patients who underwent transplantation not in complete remission (non-CR).^57^ Another study suggested that DNMT3A-mutated donor clones may enhance graft-versus-leukemia effects, particularly in the absence of post-transplant cyclophosphamide (PTCy), contributing to lower relapse rates and improved progression-free survival. Importantly, no cases of donor cell leukemia were observed in recipients of grafts with only DNMT3A mutations.^59^ These findings highlight the relatively benign role of DNMT3A compared to other CHIP variants and highlight the need for mutation-specific decision-making in donor selection.

## Screening, Therapeutic Horizons, and Ethical Considerations

Screening for CHIP in asymptomatic adults remains a debated topic. While not currently recommended due to the absence of formal guidelines and unclear clinical evidence,^60^ emerging data suggest potential value in selected contexts. Targeted testing may be considered in older adults with unexplained cytopenias, or in potential HSC donors over the age of 50 to minimize risks of donor-derived malignancy, GVHD, and cytopenias.^53,61^ Identifying commonly mutated CHIP genes can enable early recognition of individuals at elevated risk for CVD and hematologic malignancy.^62^ For CHIP-positive individuals, preventive strategies including lipid management, blood pressure optimisation, smoking cessation, healthy dietary habits, and monitoring for hematologic changes may be particularly beneficial.^49,63^ Moreover, specific driver mutations may guide personalized therapies. For instance, the IL-1β inhibitor canakinumab reduced cardiovascular events in the CANTOS trial, with post hoc analyses indicating enhanced benefit among CHIP carriers.^62,64^ Experimental and genetic studies also highlight the therapeutic potential of targeting inflammatory mediators such as AIM2 in JAK2-mutant CHIP.^31^

Newer cardiovascular risk models are moving beyond just cholesterol and blood pressure. Combining CHIP mutations, including JAK2 or TET2, inflammatory markers such as AIM2, IL-6, and more polygenic risk scores, and traditional risk factors, offers a more personalized way to predict and prevent heart disease. Large studies like the UK Biobank and MESA show that this approach can better identify people, especially those with high-risk CHIP mutations or inflammatory profiles, who may benefit from early interventions.^65^

CRISPR and lentiviral vector studies have demonstrated that inactivating mutations in DNMT3A and TET2 within the hematopoietic cells contribute to CVD by promoting RAAS-induced cardiac dysfunction, hypertrophy, fibrosis, and inflammation, with gene-specific effects on cardiac remodeling and hematopoietic cell expansion.^31^ While these findings provide a mechanistic insight, such gene editing approaches remain experimental and not clinically applicable at this time. Similarly, modifying the bone marrow niche or enhancing wild-type HSC fitness to suppress mutant clonal expansion holds theoretical promise but lacks validated, targeted interventions in humans.^4,66^

More clinically relevant strategies are also under investigation. For instance, vitamin C treatment has been shown to restore TET2 activity in deficient HSCs and reverse abnormal self-renewal in preclinical models, potentially limiting clonal expansion.^67^ In JAK2-mutant CHIP, the JAK2 inhibitor ruxolitinib has demonstrated the ability to reduce thrombosis and neutrophil extracellular trap (NET) formation via modulation of the IL-8 pathway in mouse models.^68,69^ While these pharmacologic approaches offer more immediate translational potential, further clinical trials are necessary to establish their efficacy and safety in CHIP-related CVD (**Table 1**).

**Table 1.** Therapeutic Strategies Targeting CHIP-Driven Cardiovascular Risk.

**Therapeutic Approach**	**Mechanism**	**Key Findings**	**References**
Anti-⁠Inflammatory Therapies	CHIP mutations (TET2, DNMT3A) promote IL-1β/IL-6 signaling, inflammasome activation, and vascular inflammation. Blocking these pathways reduces CVD risk.	CANTOS post-hoc trial: TET2-mutant carriers had a greater reduction in recurrent CV events with IL-1β inhibitor canakinumab. IL-6 signaling deficiency reduced CVD risk in CHIP. IL-6 blockade attenuates atherosclerosis in TET2-deficient mice.	^62^
Anti-Thrombotic	CHIP increases inflammation, endothelial activation, and pro-thrombotic risk (including pulmonary embolism, NET formation). Antithrombotic therapies may mitigate this excess risk.	Observational link between CHIP and pulmonary embolism. NET formation promotes thrombosis in myeloproliferative neoplasms, relevant to CHIP.	^21,68,69^
Conventional CVD Therapies	Standard lipid-lowering and risk-factor-modifying therapies may provide benefit in CHIP carriers. Statins may mitigate vascular inflammation; lifestyle/metabolic interventions reduce the additive risk.	Reviews emphasize the need to apply conventional therapies more aggressively in CHIP carriers. CHIP is linked to worsened outcomes in HF, AF, and diabetes.	^49,63^
Gene-editing / Experimental Approaches	Direct correction of CHIP mutations (e.g., DNMT3A, TET2) or restoration of their function could prevent expansion of mutant clones. Preclinical CRISPR studies show feasibility. TET2 restoration reverses aberrant self-renewal.	CRISPR editing of TET2/DNMT3A mutations reduced CVD burden in mice. Restoring TET2 function blocked leukemia progression.	^31^
Metabolic / Repurposed Agents	Drugs that reduce the fitness advantage of mutant HSPCs or dampen CHIP-driven inflammation may be protective.	Metformin reduced the competitive advantage of DNMT3A-mutant HSPCs in preclinical models.	^66^
Genetic modification of inflammatory risk	Mendelian randomization shows inherited IL-6R deficiency attenuates CVD risk in CHIP, highlighting host-gene interactions as potential therapeutic targets.	Genetic IL-6 signaling deficiency lowered CV risk among CHIP carriers.	^48^

The incidental discovery of CHIP poses ethical and clinical challenges. Although often asymptomatic, CHIP is associated with adverse cardiovascular outcomes, including in patients with heart failure and reduced ejection fraction.^70^ Larger clones (VAF >10%) carry significantly higher risks, such as worse outcomes after transcatheter aortic valve implantation (TAVI) and increased coronary events.^71^ However, intervening in asymptomatic CHIP carriers remains controversial due to the potential risk of overdiagnosis, unnecessary follow-up, and psychological distress from learning of a mutation with uncertain prognosis.^72^

Ethical issues include the importance of informed consent. Patients must understand the uncertain implications and limited treatment options. There are risks of genetic discrimination, and the high cost of testing and monitoring may restrict access, worsening healthcare disparities. Socioeconomic status and healthcare literacy can further impact the interpretation and management of CHIP. A cautious, patient-centered approach focused on individualized risk assessment and equitable care is essential while further research defines clear clinical pathways.^73^

## Conclusion

CHIP represents a convergence point between multiple biological systems, including aging, immunity, hematology, and vascular biology. It was traditionally confined to hematologic malignancy risk, but recent investigations have revealed its role in CVD, linking somatic mutations in hematopoietic stem cells to systemic inflammatory processes. As individuals age, mutations in genes including TET2, DNMT3A, and ASXL1 lead to an expansion of mutant clones whose effects expand beyond the bone marrow through various inflammatory pathways. These, in turn, contribute to endothelial dysfunction, accelerated atherosclerosis, and heart failure.

CHIP holds promise as both a predictive marker and a therapeutic axis for age-related CVD. The presence of CHIP mutations could allow us to refine the current cardiovascular risk prediction models, especially in older adults who lack traditional risk factors. Moreover, the proinflammatory pathways, including the 1β and NLRP3 inflammasome pathways, could serve as potential therapeutic targets. Future translational strategies, including gene-editing or small-molecule inhibitors, could suppress clonal expansion and the downstream proinflammatory pathways to mitigate the risk of CHIP-associated CVD.

Future prospective clinical trials are essential to understand and evaluate CHIP-informed intervention strategies. Such studies should assess whether targeting CHIP in at-risk individuals reduces cardiovascular events, which in turn could help improve patient stratification and guide personalized prevention strategies. Integration of single-cell sequencing and AI-driven risk models can be crucial in this personalized approach, allowing maximum reduction in CVD risk. As our population continues to age, addressing CHIP may be key to reducing the burden of cardiovascular disease and advancing precision medicine in vascular health

### Authors’ Contribution

Conceptualization: Soumiya Nadar, Taha Kassim Dohadwala

Methodology: Soumiya Nadar, Taha Kassim Dohadwala

Formal Analysis: Soumiya Nadar, Taha Kassim Dohadwala, Nitish Kumaresan

Investigation: Soumiya Nadar, Taha Kassim Dohadwala, Nitish Kumaresan

Writing – Original Draft Preparation: Soumiya Nadar, Taha Kassim Dohadwala, Nitish Kumaresan, Shabbeer Imtiaz Ahamed, Sumia Fatima

Writing – Review & Editing: Soumiya Nadar, Taha Kassim Dohadwala, Nitish Kumaresan, Shabbeer Imtiaz Ahamed, Sumia Fatima

Resources: Soumiya Nadar, Taha Kassim Dohadwala, Sumia Fatima

Supervision: Soumiya Nadar, Taha Kassim Dohadwala, Sumia Fatima

Visualization: Soumiya Nadar, Taha Kassim Dohadwala, Shabbeer Imtiaz Ahamed

Project Administration: Soumiya Nadar, Taha Kassim Dohadwala

### Competing of Interest – COPE

No competing interests were disclosed.

### Informed Consent Statement

Not Applicable.

## Data Availability

No patient data was directly used in this study.
